# A randomised controlled trial of a lifestyle behavioural intervention for patients with low back pain, who are overweight or obese: study protocol

**DOI:** 10.1186/s12891-016-0922-1

**Published:** 2016-02-11

**Authors:** Amanda Williams, John Wiggers, Kate M. O’Brien, Luke Wolfenden, Serene Yoong, Elizabeth Campbell, Emma Robson, James McAuley, Robin Haskins, Steven J. Kamper, Christopher M. Williams

**Affiliations:** Hunter New England Population Health, Locked Bag 10, Wallsend, NSW, 2287 Australia; Hunter Medical Research Institute, Locked Bag 1, Hunter Region Mc, NSW 2310 Newcastle, Australia; University of Newcastle, Newcastle, 2308 Australia; Neuroscience Research Australia, PO Box 1170, Randwick, NSW 2031 Australia; Prince of Wales Clinical School, University of New South Wales, Randwick, 2031 Australia; Ambulatory Care Centre, John Hunter Hospital, Hunter New England Local Health District, Locked Bag 664 J, Newcastle, NSW 2300 Australia; The George Institute for Global Health, University of Sydney, PO Box M201, Sydney, NSW 2050 Australia

**Keywords:** Low back pain, Obesity, Lifestyle, Telephone, Randomised controlled trial, Protocol

## Abstract

**Background:**

Low back pain is a highly prevalent condition with a significant global burden. Management of lifestyle factors such as overweight and obesity may improve low back pain patient outcomes. Currently there are no randomised controlled trials that have been conducted to assess the effectiveness of lifestyle behavioural interventions in managing low back pain. The aim of this trial is to determine if a telephone-based lifestyle behavioural intervention is effective in reducing pain intensity in overweight or obese patients with low back pain, compared to usual care.

**Methods/Design:**

A randomised controlled trial will be conducted with patients waiting for an outpatient consultation with an orthopaedic surgeon at a public tertiary referral hospital within New South Wales, Australia for chronic low back pain. Patients will be randomly allocated in a 1:1 ratio to receive a lifestyle behavioural intervention (intervention group) or continue with usual care (control group). After baseline data collection, patients in the intervention group will receive a clinical consultation followed by a 6-month telephone-based lifestyle behavioural intervention (10 individually tailored sessions over a 6-month period) and patients in the control group will continue with usual care. Participants will be followed for 26 weeks and asked to undertake three self-reported questionnaires at baseline (pre-randomisation), week 6 and 26 post randomisation to collect primary and secondary outcome data. The study requires a sample of 80 participants per group to detect a 1.5 point difference in pain intensity (primary outcome) 26 weeks post randomisation. The primary outcome, pain intensity, will be measured using a 0–10 numerical rating scale.

**Discussion:**

The study will provide robust evidence regarding the effectiveness of a lifestyle behavioural intervention in reducing pain intensity in overweight or obese patients with low back pain and inform management of these patients.

**Trial registration number:**

Australian New Zealand Clinical Trials Registry, ACTRN12615000478516, Registered 14/05/2015.

## Background

Low back pain is a common condition and poses significant burden to individuals and society. Globally, the median point prevalence of low back pain has been reported to be 15 % [1] and the global lifetime prevalence as high as 84 % [2]. The latest Global Burden of Disease Study (2013) reported over 651 million cases of low back pain in 2013, which is the leading cause of disability measured [3]. As a consequence, low back pain represents a considerable economic burden. Direct costs of care are reported to be more than $AU4.7 billion in Australia (2012 values), more than £1.6 billion in the United Kingdom (1998 values) and as much as $US90 billion in the United States (1998 values) [4, 5].

**Table 1:** Trial Measures

Outcome	Domain	Measures	Time point (weeks)
Primary	Pain intensity	Pain intensity over the previous week as measured by the 0–10 Numerical Rating Scale (NRS) [26]	0, 2, 6, 10, 14, 18, 22, 26
Secondary	Disability	Roland Morris Disability Questionnaire (RMDQ) [28]	0, 6, 26
	Self-reported weight	Self-reported weight (kg)	0, 6, 26
	Objective weight	Measured to the nearest 0.1 kg [29]	0^a^, 26
	BMI	Calculated as weight/height squared (kg/m^2^)	0^a^, 26
	Waist circumference	Measured to the nearest 0.1 cm [29]	26
	Quality of life	Short Form 12 version 2 (SF12.v2) [30]	0, 6, 26
	Perceived change in condition	Global Perceived Effect Scale [43]	6, 26
	Psychological distress	Depression, Anxiety and Stress Scale-21 (DASS-21) [31]	0, 26
	Sleep quality	Item 6 of the Pittsburgh sleep quality index [32]	0, 6, 26
	Health behaviours	Physical Activity measured using the Active Australia Survey [33]	0, 6, 26
		Dietary intake measured using a short food frequency questionnaire [34]	0, 6, 26
		Alcohol Consumption measured using the alcohol use disorders identification test (AUDIT) [35]	0, 6, 26
		Self-reported smoking status [36]	0, 6, 26
	Health care utilisation	Medication use for low back pain	0, 6, 26
		Visits for low back pain – type and number of sessions	0, 6, 26
		Attended orthopaedic consultation, received surgery	26
	Pain attitudes	Survey of Pain Attitudes (SOPA) [38]	0, 6, 26
	Fear Avoidance	Fear Avoidance Beliefs Questionnaire (FABQ) [39]	0, 26
	Economic	Quality of life (SF12.v2)	0, 6, 26
		Health care utilisation (including estimated out of pocket cost)	
		Absenteeism (days off normal work due to lower back pain in the past 6 weeks)	

*GHS*: Get Healthy Information and Coaching Service; ^a^Intervention group only

While the aetiology of low back pain remains unclear, it is now widely accepted that effective treatment for low back pain requires consideration of the psychological and behavioural factors. Several lifestyle behavioural factors are reported to be associated with an increased prevalence and persistence of low back pain including weight, sleep disturbance, psychological distress, and beliefs. Among the most compelling evidence is the association between overweight and obesity and low back pain [6, 7]. One meta-analysis which included 33 cross-sectional and cohort studies, found significant associations between overweight or obesity and a range of low back pain outcomes. Data from the cohort studies showed that overweight or obesity is associated with an increased 12-month prevalence of low back pain (*n* = 6828; OR 1.21, 95 % CI: 1.07, 1.37), increased risk of chronic low back pain (defined as longer than 3 months in duration; OR 1.43, 95 % CI: 1.28, 1.60), and higher rates of health care seeking for low back pain (OR 1.56, 95 % CI: 1.46, 1.67) [7]. Similar associations have been reported for body mass index (BMI) [8]. While the association between physical activity and diet and low back pain is less consistent, these are key drivers of weight gain [9]. Certainly, patients with low back pain who are overweight or obese, are likely to have more complex health needs requiring focus on a holistic lifestyle and behavioural approach to management.

Given these widely reported associations between lifestyle behavioural factors and low back pain, it is suggested that targeting these as part of low back pain management could improve patient outcomes [7, 10, 11]. While international guidelines for weight management recommend behavioural modification interventions as the preferred approach to managing weight loss and healthy lifestyle there is limited evidence to guide such care in patients with low back pain [9]. Several systematic reviews have found no randomised controlled trials (RCT) reporting the effectiveness of lifestyle behavioural interventions in managing persistent low back pain [10, 12]. To the author’s knowledge only one pre-post study of a 52 week medically supervised weight loss program for obese patients with low back pain has been conducted. The study found a statistically significant weight loss of 15.3 kg (95 % CI: 7.8, 22.8) was associated with a significant improvement in pain related disability (Oswestry Disability Index (ODI) baseline 31.9 ± 17.7, follow-up 27.1 ± 20.9, *p* = 0.009) [13]. While promising, there is a need to test the effectiveness of lifestyle behavioural interventions on low back pain outcomes in robust RCTs.

Given the large numbers of patients who suffer from low back pain and are overweight or obese [6, 7], an important consideration is to provide cost effective interventions that are accessible to a large proportion of overweight patients at relatively lower cost to patients. Telephone-based interventions as a treatment delivery modality has potential to provide greater access to treatment for patients, and overcomes barriers to accessing continued care, including time and travel requirements to attend face-to-face appointments, and flexible scheduling of contact [14]. Importantly, telephone-based interventions that include behavioural modification and adjunct psychological strategies are consistently shown to be as effective as face-to-face interventions in achieving weight loss [15, 16]. For the key determinants of weight loss; physical activity and diet modification, telephone-based interventions have also been shown to be more cost-effective compared to clinical face-to-face practices [14].

The primary objective of the study is to determine if a telephone-based lifestyle behavioural intervention is effective in reducing pain intensity in overweight or obese patients with low back pain, compared to usual care. Secondary objectives are to investigate if the intervention improves key secondary outcomes: disability and function, anthropometry (weight, BMI, waist circumference), quality of life, diet, physical activity and health care utilisation, compared to usual care.

## Methods

### Study design and setting

**Fig. 1:**
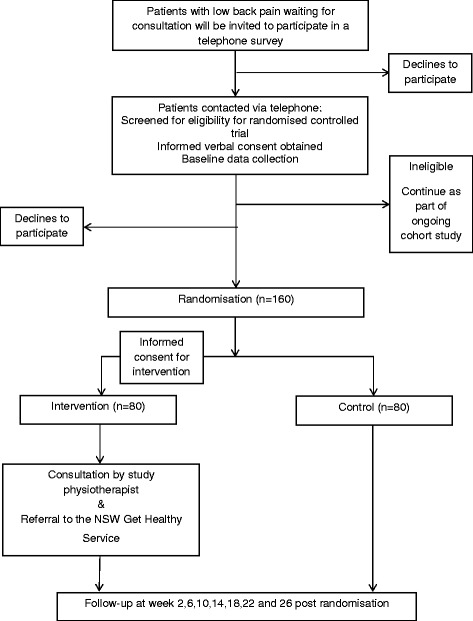
Progress of participants through the study

The study will employ a parallel group randomised controlled design (Fig. 1), as part of a cohort multiple RCT [17]. This pragmatic design utilises participants from our existing cohort of routine service; patients are randomised to be offered a new clinical intervention (intervention group) or to remain part of the cohort (control group). The control group is not aware of the intervention trial and thus act as a real world usual care comparison. This protocol adheres to the requirements of the Standard Protocol Items: Recommendations for Intervention Trials (SPIRIT) guidelines and is prospectively registered with the Australian New Zealand Clinical Trials Registry (ACTRN12615000478516). The trial will be undertaken in the Hunter New England Local Health District, New South Wales (NSW), Australia. Ethical approval has been obtained from the Hunter New England Human Research Ethics Committee (approval No. 13/12/11/5.18) and the University of Newcastle Human Research Ethics Committee (approval No. H-2015-0043).

### Population and recruitment

One hundred and sixty patients waiting for an outpatient orthopaedic consultation at a public tertiary referral hospital within NSW for non-specific low back pain will be recruited. All patients over 18 years of age waiting for an outpatient consultation for low back pain will be sent an information letter to invite participation in a telephone survey as part of the ongoing cohort study. Patients will be asked to contact the researchers if they do not wish to participate or can refuse upon receipt of the telephone call. Patients consenting to the telephone survey will then be screened for eligibility for the RCT by a trained interviewer, invited to participate if eligible for the study and asked to complete the baseline survey at the time of the call.

To be eligible participants must meet the following criteria:

- Chronic low back pain defined as: pain in the lower back (i.e. between the 12th rib and buttock crease) with/without leg pain and a duration of longer than 3 months since the onset of pain [18];
- Aged 18 years or older;
- Classified as overweight or obese with a BMI of ≥27 kg/m^2^ and <40 kg/m^2^ – based on self-reported weight and height;
- Have access to and can use a telephone;
- Low back pain severe enough to cause at least average low back pain intensity ≥3 of 10 on a 0–10 numerical rating scale (NRS) in the last week or moderate level of interference in activities of daily living (adaptation of item 8 on SF36).

Patients will be excluded if they meet the following criteria:

- Known or suspected serious pathology as the underlying cause of back pain (e.g. fracture, cancer, infection, inflammatory arthritis, cauda equine syndrome);
- A previous history of obesity surgery;
- Currently participating in any prescribed, medically supervised or commercial weight loss program;
- Back surgery in the last 6 months or booked in for surgery in the next 6 months;
- Unable to comply with the study protocol that requires them to, adapt meals or exercise, due to non-independent living arrangements;
- Any medical or physical impairment, apart from back pain, precluding safe participation in exercise such as uncontrolled hypertension, or morbid obesity (BMI ≥40);
- Cannot speak and read English sufficiently to complete the study procedures.

### Randomisation and blinding

A randomisation schedule will be created a priori by an independent investigator using SAS 9.3 through the SURVEYSELECT procedure. Consenting patients who are eligible for the trial will be allocated, in a 1:1 allocation ratio, to either receive the lifestyle behavioural intervention at that time (intervention group) or remain as part of the cohort and be told they will be offered clinical services in 6 months (control group). To randomise patients, a trained interviewer will open a sealed opaque envelope containing group allocation. A staff member not involved in the study will prepare the envelopes. Patient progress through the study is outlined in Fig. 1.

All outcome assessors will be blind to group allocation.

### Treatments

#### Intervention group

Patients randomised to the intervention group will be provided brief advice and education about the benefits of weight loss and physical activity for their conditions by trained telephone interviewers. Participants will then be invited to attend a one hour consultation with the study physiotherapist at Hunter New England Population Health, NSW, Australia and referred to the NSW Get Healthy Information and Coaching Service (GHS) [19, 20].

#### Consultation

The consultation will involve a low back pain clinical assessment and detailed low back pain education based on principles recommended by clinical practice guidelines. The consultation will also apply behaviour change techniques to support a healthy lifestyle and weight management for low back pain. This intervention content was informed by Self Determination Theory (SDT) [21, 22]. According to SDT autonomous behaviour rather than behaviour controlled by rewards, punishments or self-imposed pressures is more likely to result in long lasting behaviour change [22]. The constructs deemed integral in SDT to develop autonomous motivation include increasing 1) perceived competence (increase interest, enjoyment and importance) and 2) self-regulation (increase ability to direct behaviour to act in your long term best interest and in line with your values) [22]. The specific techniques used in the consultation to address these key constructs include: i) provision of education and reassurance to correct inappropriate pain beliefs and improve self-efficacy for self-management (i.e. provide information about the about the nature of the condition, that persistent low back pain is multifactorial with multiple influences and not usually the result of pathological damage), ii) acknowledging the consequences of unhealthy lifestyle factors (overweight, inactivity, poor diet, alcohol misuse, smoking, poor sleep) on low back pain, iii) provide general encouragement and examples of how improving lifestyle factors can influence pain outcomes and quality of life, iv) prompt commitment from the participant, v) acknowledge that monitoring of behaviours will be conducted throughout the program, vi) setting graded tasks to adopt better physical functioning and healthy behaviour (e.g. begin walking 30 min daily), vii) encourage self-monitoring of goals, viii) present the NSW GHS as a way to support ongoing behaviour change to improve low back pain and general health, ix) acknowledge general barriers that may reduce motivation to change lifestyle and adherence to the program (e.g. acknowledge fluctuating nature of condition and that high levels of pain are the result of a complex interaction of factors not just the result of increased activity, and discourage use of pain as a guide for progression of activity).

#### Lifestyle behavioural intervention - The NSW Get Healthy Service

Following the consultation, patients randomised to the intervention group will be referred to the established GHS [19]. The referral to the GHS will be provided to the service on the participants’ behalf. The GHS is a free telephone-based government funded service to support individuals to modify their eating behaviours, increase their physical activity, reduce alcohol consumption and maintain a healthy weight or reduce their weight. The service was developed in response to evidence supporting the efficacy of telephone-based behaviour modification interventions and facilitates the translation of this evidence into a population wide approach [19]. A pre-post study assessing the effectiveness of the GHS in the general population reported significant reductions in weight, BMI, and waist circumference, and significant improvements in physical activity and nutrition-related behaviours [19].

The GHS service involves 10 individually tailored coaching calls delivered over a 6 month period by a qualified health professional including dieticians, exercise physiologists and psychologists [19]. The support provided is based on national guidelines including the Australian Guide to Healthy Eating and National Physical Activity Guidelines [19, 23], utilises motivational interviewing principles [19, 24], addresses health-related psychological blocks with Socratic questioning [25], and applies self-regulation principles including goal setting, overcoming barriers and creating sustainable changes [19]. The program is individually tailored to each patient with content targeted to address individual patient goals throughout the 6 months and phone calls scheduled according to the patient’s preferences. These aspects are determined by the patient and health coach together, however calls are generally provided on a tapered schedule, with a higher intensity of calls (*n* = 6) made within the first three months of the program [19]. This schedule facilitates initiation of behaviour change in the first three months and maintenance and prevention of relapse in the second half of the program. In addition to the health coaching calls, participants receive an information booklet that provides additional information to support them during the program to achieve their goals, a coaching journal to record goals and actions, and access to online services to help track their progress. Medical clearance from a general practitioner will be obtained when required, as per existing service protocols [19].

All health coaches, regardless of multidisciplinary background, receive training to ensure they meet the requirements of the service and to promote consistency across the program. The service conducts audits of coaching quality as part of its quality improvement practices. To ensure the GHS health coaching is relevant for low back pain participants, health coaches will be provided additional training by a study investigator (CW) in evidence-based management for low back pain (2 h interactive training session) and provided with information resources to guide specific advice to be provided to study participants. The training session includes the topics of diagnosis, prognosis and evidence-based management strategies including the role of a healthy lifestyle and weight loss. The information provided is contained within international clinical practice guidelines for low back pain. Resources also detail guideline recommended advice about the nature of the condition, the diagnosis, prognosis and evidence-based treatments, as well as common misconceptions about back pain and its management.

#### Control group

Participants randomised to the control group will continue on the usual care pathway and take part in data collection during the 6 month intervention period. Currently no active management of low back pain patients waiting for an outpatient orthopaedic consultation occurs. Control group patients will be informed that a face-to-face appointment to determine the need for further care will be available in 6 months.

### Data collection

Participants will be followed for 6 months (26 weeks) and be asked to complete three self-reported questionnaires at baseline (pre-randomisation), week 6 and 26 weeks post randomisation to collect primary and secondary outcome data. All participants will be mailed a questionnaire one week prior to the 6 and 26 week time point and then asked to provide responses in one of two ways: via telephone or returned postal questionnaire. The baseline questionnaire will be completed via telephone only. Participants will also be asked to record the primary outcome ‘pain intensity’ at week 2, 10, 14, 18 and 22. Participants will be asked to provide these data via telephone or reply to text message, whichever their preference. During the 26 week telephone survey participants will be asked to attend a follow up clinic appointment (intervention group) or initial clinical appointment (control group) with a health professional.

### Measures

#### Baseline demographic characteristics

The following demographic items will be collected at baseline: age, Aboriginal and/or Torres Strait Islander status, employment status, country of origin, highest level of education, health insurance status and medical conditions. Length of time waiting for consultation (days) and triage classification will be obtained from hospital records.

#### Primary outcome

##### Pain intensity

Pain intensity will be measured using a 0–10 NRS, as the average pain over the last week where zero indicates ‘no pain’ and ten indicates the ‘worst possible pain’ [26]. Pain intensity will be collected at baseline, at 2, 6, 10, 14, 18, 22 and 26 weeks post randomisation (see Table 1). The NRS is a valid and reliable measure of pain intensity in adults with low back pain [27].

#### Secondary outcomes

The secondary outcomes include: low back pain disability, using the Roland Morris Disability Questionnaire (RMDQ) [28]; self-reported weight (kg); objective weight (kg) measured to the nearest 0.1 kg by a trained assessor using International Society for the Advancement of Kinanthropometry (ISAK) procedures [29]; BMI calculated as weight /height squared (kg/m^2^); waist circumference measured at 26 weeks post randomisation taken at the level of the narrowest point between the inferior rib border and the iliac crest by trained assessors using a flexible tape measure to the nearest 0.1 cm [29]; quality of life assessed using the 12-item Short Form Health Survey version 2 (SF-12.v2) [30]; global perceived change in condition measured using the Global Perceived Effect Scale (−5 to 5 scale) [29]; psychological distress using the Depression, Anxiety and Stress Scale-21 (DASS-21) [31]; sleep quality measured using item 6 of the Pittsburgh Sleep Quality Index [32]; health behaviours including physical activity reported as the frequency and total minutes of spent participating in physical activity measured by the Active Australia Survey [33], dietary intake measured by a short food frequency questionnaire (FFQ) [34], alcohol consumption measured using the Alcohol Use Disorders Identification Test (AUDIT) [35] and self-reported current smoking status [36]; health care utilisation including medication use, type of health services utilised for low back pain and the number of sessions [37]; and attitudes and beliefs measured by the Survey of Pain Attitudes (SOPA) [38] and the Fear Avoidance Beliefs Questionnaire (FABQ) [39]. See Table 1 for data collection time points for secondary outcomes. 

### Intervention and data integrity

The delivery of the intervention will be assessed using attendance records for the physiotherapy consultation and data regarding delivery of the GHS intervention including, commencement and number, length, timing of coaching calls and achievement of identified goals which will be provided by the GHS. Patient reported receipt of care (as well as additional care) will be collected at all secondary collection time points. Participants will be monitored for adverse events throughout the intervention period. All adverse events will be recorded and serious adverse events will be assessed and managed on a case-by-case basis according to Good Clinical Practice (GCP) guidelines [40]. Trial data integrity will be monitored by regularly scrutinising data files for omissions and errors. Manually entered data (i.e. data not recorded directly by participant) will be double entered and the source of any inconsistencies will be explored and resolved in consultation with the lead investigator (CW). Data will be stored on password protected files, with access given to approved researchers only.

### Sample size

Sample size was calculated using Stata sample size calculator. Using a standard deviation of 2.3, a two-sided alpha of 0.025 (to account for multiple outcomes of interest – pain and weight) [41] and allowing for 15 % loss to follow up, a sample of 80 participants per group will provide 90 % power to detect a clinically meaningful difference of 1.5 in pain intensity (pain numerical rating scale) between intervention and control groups at 26 weeks post randomisation. This sample also provides power 80 % to detect a 6 % reduction in weight in the underlying sampling population and based on evidence from other musculoskeletal conditions is hypothesized to lead to a clinically meaningful reduction in pain [42].

### Statistical analysis

#### Primary outcomes analysis

Between group differences in pain intensity will be assessed using linear mixed models, with random intercepts for individuals to account for correlation of repeated measures. We will obtain estimates of the effect of the intervention and 95 % confidence intervals by constructing linear contrasts to compare the adjusted mean change in outcome from baseline to each time point between the treatment and control groups. Dummy coded variables representing group allocation will be used to ensure blinding of the analyses. Missing data will be assessed for randomness if this is more than 10 %.

#### Secondary outcomes analysis

Linear mixed models will be used to assess treatment effects on secondary outcomes as per the primary outcome. We will compare the adjusted mean change (continuous variables) or difference in proportions (dichotomous variables) in outcome from baseline to each time point between the treatment and control groups.

An economic evaluation will also be undertaken. We will develop costing models from the perspective of the health service and broader societal perspective. These models will utilize data regarding patient quality of life (SF12v2), health care and community services use, work absenteeism. We will calculate costs based on published normative data and estimated out of pocket costs reported by participants. We will also investigate the mechanisms underlying the intervention using causal mediation analysis and include the following measures at baseline, 6 weeks and 6 months: pain attitudes (SOPA), fear avoidance beliefs (FABQ) and symptoms of psychological distress (DASS 21), weight loss (kg), health behaviours (physical activity (MVPA), diet, alcohol, smoking, sleep quality).

## Discussion

This is the first RCT designed to evaluate the effectiveness of a lifestyle behavioural intervention for low back pain patients who are overweight or obese. The results will inform care pathways by providing robust evidence about the effectiveness of such management for overweight patients with low back pain.

## Abbreviations

BMI: Body Mass Index
RCT: Randomised controlled trial
ODI: Oswestry Disability Index
SPIRIT: Standard Protocol Items: Recommendations for Intervention Trials
NSW: New South Wales
NRS: Numerical rating scale
GHS: NSW Get Healthy Information and Coaching Service
RMDQ: Roland Morris Disability Questionnaire
ISAK: International Society for the Advancement of Kinanthropometry
SF12.v2: Short Form Healthy Survey version 2
DASS-21: Depression Anxiety Stress Scale-21
FFQ: Food Frequency Questionnaire
AUDIT: Alcohol Use Disorders Identification Test
SOPA: Survey of Pain Attitudes
FABQ: Fear Avoidance Beliefs Questionnaire
GCP: Good Clinical Practice
HMRI: Hunter Medical Research Institute
